# Sirtuin1 Over-Expression Does Not Impact Retinal Vascular and Neuronal Degeneration in a Mouse Model of Oxygen-Induced Retinopathy

**DOI:** 10.1371/journal.pone.0085031

**Published:** 2014-01-08

**Authors:** Shaday Michan, Aimee M. Juan, Christian G. Hurst, Zhenghao Cui, Lucy P. Evans, Colman J. Hatton, Dorothy T. Pei, Meihua Ju, David A. Sinclair, Lois E. H. Smith, Jing Chen

**Affiliations:** 1 Instituto Nacional de Geriatría, Institutos Nacionales de Salud, México; 2 Department of Ophthalmology, Boston Children's Hospital, Harvard Medical School, Boston, Massachusetts, United States of America; 3 Manton Center for Orphan Disease Research, Boston Children's Hospital, Harvard Medical School, Boston, Massachusetts, United States of America; 4 Paul F. Glenn Laboratories for the Biological Mechanisms of Aging, Department of Genetics, Harvard Medical School, Boston, Massachusetts, United States of America; 5 Department of Pharmacology, The University of New South Wales, Kensington, Australia; Cedars-Sinai Medical Center; UCLA School of Medicine, United States of America

## Abstract

Proliferative retinopathy is a leading cause of blindness, including retinopathy of prematurity (ROP) in children and diabetic retinopathy in adults. Retinopathy is characterized by an initial phase of vessel loss, leading to tissue ischemia and hypoxia, followed by sight threatening pathologic neovascularization in the second phase. Previously we found that Sirtuin1 (Sirt1), a metabolically dependent protein deacetylase, regulates vascular regeneration in a mouse model of oxygen-induced proliferative retinopathy (OIR), as neuronal depletion of Sirt1 in retina worsens retinopathy. In this study we assessed whether over-expression of Sirtuin1 in retinal neurons and vessels achieved by crossing Sirt1 over-expressing flox mice with Nestin-Cre mice or Tie2-Cre mice, respectively, may protect against retinopathy. We found that over-expression of Sirt1 in Nestin expressing retinal neurons does not impact vaso-obliteration or pathologic neovascularization in OIR, nor does it influence neuronal degeneration in OIR. Similarly, increased expression of Sirt1 in Tie2 expressing vascular endothelial cells and monocytes/macrophages does not protect retinal vessels in OIR. In addition to the genetic approaches, dietary supplement with Sirt1 activators, resveratrol or SRT1720, were fed to wild type mice with OIR. Neither treatment showed significant vaso-protective effects in retinopathy. Together these results indicate that although endogenous Sirt1 is important as a stress-induced protector in retinopathy, over-expression of Sirt1 or treatment with small molecule activators at the examined doses do not provide additional protection against retinopathy in mice. Further studies are needed to examine in depth whether increasing levels of Sirt1 may serve as a potential therapeutic approach to treat or prevent retinopathy.

## Introduction

Pathologic blood vessel proliferation is a leading cause of blindness in proliferative retinopathy. This includes retinopathy of prematurity (ROP), a complication of premature birth in children, and diabetic retinopathy (DR), one of the most common complications of diabetes in working age adults [1], [2]. Finding treatment options for retinopathy, which is critical for preventing blindness in children and adults, depends on a better understand of the disease pathogenesis. Retinopathy develops in two phases. The first phase is vessel loss or regression of existing vessels after premature birth in ROP, or due to abnormal metabolism in diabetes in DR. Retinal vessel loss induces tissue ischemia and hypoxia, leading to upregulation of angiogenic growth factors, which then stimulate sight-threatening pathologic neovascularization[2]–[8]. Recent research has paid increasing attention to the interaction between retinal neurons and vessels in the pathogenesis of retinopathy[9]–[11]. Understanding how retinal neurons respond to ischemic insults and metabolic stress in retinopathy to regulate vessel growth is important for the identification of new potential therapies.

Previously we found that the presence of neuronal Sirtuin1, a metabolically dependent protein deacetylase, is essential in mediating neurovascular crosstalk and regulating vascular regeneration in a mouse model of oxygen-induced retinopathy (OIR), which mimics ROP and some aspects of proliferative DR in humans[12], [13]. Sirt1 belongs to a family of class III histone deacetylases, originally discovered to be important for calorie restriction and longevity[14]. The activity of Sirt1 is dependent on NAD+ as a co-activator[15]. Sirt1 is usually localized in the nucleus, although it may also translocate to the cytoplasm, and catalyzes deacetylation of various protein targets in addition to histones[16]. Many of these Sirt1 substrates are transcription factors such as PGC1α, FOXO, and HIF, whose activities are altered by Sirt1 mediated deacetylation, leading to changed expression of target genes[16]–[18]. Sirt1 has been associated with a variety of biologic processes including oxidative stress, gene silencing, and DNA repair, in addition to senescence, neurogenesis, circadian rhythms, neuroendocrine signals and dendritic branching, all of which impact the nervous system during normal physiologic functioning as well as with aging, and during pathologic processes[19]. Within the brain Sirt1 is indispensable for normal cognitive function[20] and its activation protects against neurodegenerative diseases including Parkinson's, Alzheimer's and Huntington's disease in animal models[21]–[23]. In addition to its role in neurons, Sirt1 also plays a role in blood vessel growth during development through regulation of Notch signaling [24], and FOXO1[25]. We found in a previous study that Sirt1 is upregulated in retinal ganglion cells in the vaso-obliteration zone in ischemic neuronal retina, and conditional depletion of Sirt1 in retinal neurons significantly impaired vascular regrowth into the avascular zone and precipitated pathologic neovascularization in retinopathy[12]. These results suggest that Sirt1 is a critical stress-induced metabolically dependent protector in retinopathy.

In this study we investigated whether upregulation of Sirt1 levels or activity via genetic and pharmacologic approaches might protect against retinopathy in a mouse model of OIR. Conditional Sirt1 over-expression in retinal neurons or vessels was generated by breeding Sirt1 over-expressing flox mice with Nestin-Cre or Tie2-Cre mice, respectively. In addition, small molecule Sirt1 activators resveratrol and SRT1720[26], [27] were orally supplemented in wild type mice before and during induction of retinopathy to evaluate their potential effects. No significant protection was observed with either over-expression of Sirt1 in transgenic mice or with treatment of Sirt1 activators. These data suggest that although induction of endogenous Sirt1 in ischemic retina is critical under stress condition to protect against retinopathy, over-expression of Sirt1 or treatment with small molecule activators at the examined doses does not offer additional protective effects in retinopathy in mice.

## Methods

### Animals

These studies were approved by the Children's Hospital Boston Animal Care and Use Committee. Sirt1 over-expression mice were previously described[20]. Nestin-Cre mice (stock# 003771), Tie2-Cre mice (stock# 004128), and C57Bl/6J mice (stock# 000664) were obtained from Jackson Laboratory.

### Oxygen-induced retinopathy

Neonatal mice with their nursing mother were exposed to 75% oxygen from P7 to P12 to induce retinopathy (OIR)[13]. After returning to room air for 5 days, mice were sacrificed at P17, to observe maximal retinal neovascular response.

### Resveratrol and Sirt1 activator treatment

A micronized formulation of resveratrol, SRT501 (400 mg/kg body weight in 10 µL of 2% HPMC, 0.2% DOSS, 1% sucrose in water) or vehicle control was given daily from postnatal day (P) 5 to P17 by oral gavage to C57BL/6J mouse pups with induced OIR. Similarly, SRT1720 (100 mg/kg body weight) or vehicle control was given daily from P5 to P17 by oral gavage to C57BL/6J mouse pups with OIR. Littermate pups were used in all experiments for vehicle controls. SRT501 and SRT1720 were provided by Sirtris Pharmaceuticals, Inc. (Cambridge, MA).

### Retina dissection, staining and imaging

Mice were collected at P17 in OIR. They were anesthetized with Avertin (Sigma-Aldrich) and sacrificed via cervical dislocation. Eyes were enucleated, fixed in 4% paraformaldehyde in PBS for 1 h, and dissected to isolate the retina. The retinas were subsequently stained overnight with fluoresceinated *Griffonia Simplicifolia* Isolectin B_4_ (Alexa Fluor 594 conjugated; I21413; Invitrogen; 1∶100 dilution) in PBS with 1 mM CaCl_2_ to visualize vessels. After 2 hr washes in PBS, retinas were whole-mounted with the photoreceptor side down onto Superfrost/Plus microscope slides (12-550-15; Fisher Scientific) using SlowFade Antifade reagent (S2828; Invitrogen). Whole-mounted retinas were imaged at 5× magnification on a Zeiss AxioObserver.Z1 microscope and merged using AxioVision 4.6.3.0 software to produce images of entire retinal vasculature.

### Quantification of vessel loss and neovascularization in OIR

Vascular loss and neovascularization in OIR were quantified as previously described, using Adobe Photoshop or ImageJ[28]–[31]. By staining with Isolectin, the number of pixels in vascularized areas was visualized, outlined in Photoshop, and compared to the total number of pixels of the entire retina. From their abnormally aggregated morphology that is distinctly different from the normal branched vascular network, pathologic neovascular tuft structures were visually identified. Pixel area of pathologic neovascular tufts were quantified and compared to the total pixel area in whole retina using the SWIFT_NV method, which consists of a set of macros on NIH's free ImageJ platform to distinguish and isolate neovascular structures from the background fluorescence of normal vessels[30]. Quantification of retinal vessels was performed in a masked manner for the identity of samples. N is the number of eyes quantified.

### Quantification of retinal thickness in OIR

For retinal thickness measurements, eyes from P17 normoxia or OIR exposed animals were enucleated, fixed in 4% paraformaldehyde in PBS at room temperature for 1 h, dehydrated with increasing concentrations of ethanol and Xylene, followed by paraffin embedding. 10 paraffin sections close to the optic nerve were collected for each eye followed by H&E staining. For each retinal section, two peripheral retinal and two central retinal images were taken at 10× magnification on a Zeiss AxioObserver.Z1 microscope. Retinal thickness in cross sections of eyes was quantified in Adobe Photoshop. Using the Line Tool, the number of pixels across retina was measured for each image and retinal thickness was averaged for peripheral and central regions of each group. Evaluation was performed in a masked manner for the identity of samples. N is the number of eyes evaluated.

### Retinal RNA isolation and gene expression analysis

Total RNA was extracted from retinas of at least 3 mice. Using a mortar and pestle, retinas were lysed and subsequently filtered through QiaShredder columns (Qiagen, Chatsworth, MD, USA). RNA was extracted using the manufacturer's instructions for the RNeasy Kit (Qiagen). To generate cDNA, 1 µg total RNA was first treated with DNase I (Ambion Inc.) to remove any contaminating genomic DNA. Then, the RNA was reverse transcribed using random hexamers and Superscript III reverse transcriptase (Invitrogen Corp., Carlsbad, CA, USA). All cDNA samples were aliquoted and stored at −80°C.

The following primers were designed using Primer Bank and NCBI Primer Blast Software: mouse *Sirt1* (F: 5′-GACGATGACAGAACGTCACAC, R: 5′-CGAGGATCGGTGCCAATCA), *MAO-A* (F: 5′-GTGAATGTCAATGAGCGTCTAGT, R: 5′-TCAACAGGGATCTCTTTTCCCA), *PGC-1α* (F: 5′-GGAGCCGTGACCACTGACA, R: 5′- TGGTTTGCTGCATGGTTCTG), and *cyclophilin A* (F: 5′-CAGACGCCACTGTCGCTTT, R: 5′- TGTCTTTGGAACTTTGTCTGCAA). ABI Prism 7700 Sequence Detection System (TaqMan) and SYBR Green Master mix kit were used for the quantitative analysis of gene expression. Standard curves for each gene were plotted with a quantified cDNA template during real-time PCR reactions. The mRNA copy number of each target gene was normalized to a million copies of the housekeeping gene, *cyclophilin A*.

### Western blot

Retinal lysate was loaded on a SDS-PAGE gel and transferred onto a PVDF membrane. The membranes were blocked, incubated overnight with primary antibody, and then incubated for one hour at room temperature with secondary antibody conjugated with horseradish peroxidase. Chemiluminescence signals were generated with ECL plus substrate and captured with KODAK film. The following primary antibodies were used for Western Blot: anti-Sirt1 (Millipore, 07–131), anti-β-actin (Sigma-Aldrich, A1978); secondary antibodies: donkey anti-rabbit conjugated with horse-radish peroxidase (NA934V, Amersham Pharmacia), sheet anti-mouse conjugated with horse-radish peroxidase (NA931V, Amersham Pharmacia).

### Immunohistochemistry

Eyes were fixed in 4% paraformaldehyde in PBS for 1 h, incubated in 30% sucrose at 4°C, and embedded in OCT. 10-µm thick cross sections were collected and blocked with PBS with 0.1% Triton X-100 and 5% goat serum. Sections were stained with primary anti-Sirt1 antibody (Millipore, 07-131) and secondary antibody staining (chicken anti-rabbit Alexa 488, A-21441, Invitrogen), followed by mounting medium with DAPI.

### Statistics

Results were presented as means ± SEM and were analyzed using a two-tailed student t test. P value ≤0.05 is considered statistically significant.

## Results

### Generation of transgenic mice with conditional over-expression of Sirt1 in retinal neurons

**Figure 1:**
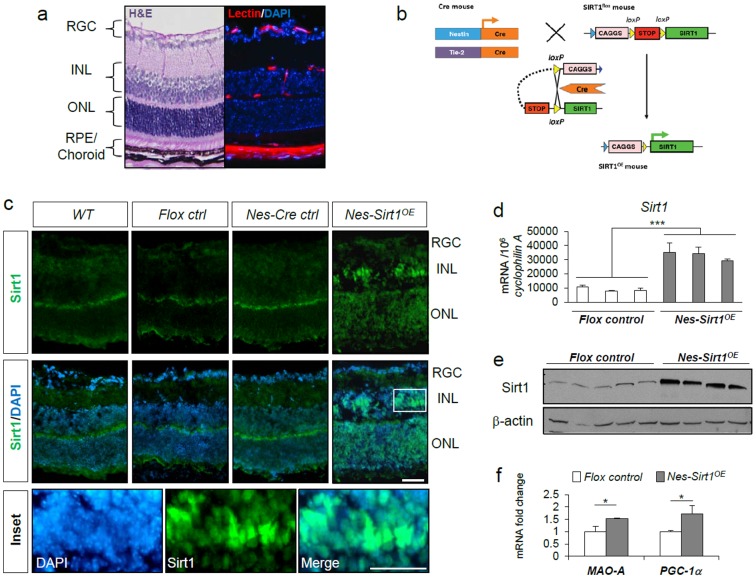
Sirt1 over-expression in retinal neurons of *Nes-Sirt1^OE^* mice. a) Representative images of retinal cross-section with: (Left) hematoxylin and eosin (H&E) staining showing three retinal neuronal layers: RGC, INL, ONL, and retinal pigment epithelium (RPE)/choroid; and (Right) lectin (red) and DAPI (blue) staining showing retinal vasculature. RGC: Retinal ganglion cell, INL: inner nuclear layer, ONL: outer nuclear layer. b) Neuronal or endothelial Sirt1 over-expressing mice (*Nes-Sirt1^OE^ or Tie2-Sirt1^OE^*) were generated by crossing Sirt1 over-expressing flox mice with Nestin-Cre or Tie2-Cre mice to result in over-expression of Sirt1 in Nestin or Tie-2 expressing cells. c) Increased nuclear Sirt1 levels were observed with immunohistochemistry in retinal sections from *Nes-Sirt1^OE^* mice compared to those from wild type (WT), flox control, Nestin-Cre controls. Sections were stained with Sirt1 antibody (green) and counter stained with nuclear stain DAPI (blue). An area in *Nes-Sirt1^OE^* retina is enlarged for visualization of Sirt1 localization in nuclei. Scale bar: 50 µm. d) Sirt1 mRNA levels, measured by RT-qPCR, were significantly increased in the retinas of postnatal day (P) 17 *Nes-Sirt1^OE^* mice compared to littermate flox controls (n = 3/group). e) Western blot analysis showed Sirt1 proteins levels were strongly increased in adult retinas of *Nes-Sirt1^OE^* mice compared to controls (n = 4–5/group). f) Increased expression of *MAO-A* and *PGC-1α* in *Nes-Sirt1^OE^* retinas compared to *flox control* retinas (n = 3/group). * p<0.05, ***p<0.001.

To evaluate the effects of increased Sirt1 levels in the retina, we generated neuronal or vascular endothelial specific Sirt1 over-expression mice (*Nes-Sirt1^OE^* and *Tie2-Sirt1^OE^*) by crossing Sirt1 flox mice (*Sirt1^flox/flox^*) with Nestin-Cre mice (*Nestin-Cre^+/−^*) or Tie2-Cre mice (*Tie2-Cre^+/−^*) expressing Cre recombinase in Nestin or Tie2 positive cells, respectively (**Fig. 1a,b**). Over-expression of Sirt1 was confirmed with Sirt1 antibody staining in retinal cross sections from WT control, flox control (*Sirt1^flox/flox^*), Nestin-Cre (*Nestin-Cre^+/−^*), and Sirt1 OE mice (*Nes-Sirt1^OE^*) (**Fig. 1c**). While all three control groups showed basal levels of Sirt1 staining in the retinas, substantially stronger nuclear staining of Sirt1 was observed in *Nes-Sirt1^OE^* retinas, particularly in the inner nuclear layer (INL). Levels of retinal *Sirt1* mRNA were quantified with RT-qPCR, showing ∼4 fold upregulation of *Sirt1* in *Nes-Sirt1^OE^* retinas at P17 compared to littermate flox controls (**Fig. 1d**). A marked increase in Sirt1 protein levels was also detected in *Nes-Sirt1^OE^* retinas compared to control retinas by Western blotting (**Fig. 1e**). In order to confirm that increased Sirt1 correlates with the activation of target genes, we analyzed expression of monoamine oxidase A (*MAO-A*), which is regulated through Sirt1-dependent deacetylation of the brain-specific helix-loop-helix transcription factor (NHLH2) to mediate anxiety and exploratory drive [32]. We also measured the well-known Sirt1 substrate *PGC-1α* [17], [33] which was previously shown to be down regulated in Sirt1-deficient retinas [12]. Our results show increased expression levels of *MAO-A*, and *PGC-1α* in *Nes-Sirt1^OE^* retinas, confirming the upregulation of Sirt1-mediated pathways in retinas that overexpress this sirtuin (**Fig. 1f**).

### Neuronal over-expression of Sirt1 does not influence vaso-obliteration and pathologic neovascularization in OIR

**Figure 2:**
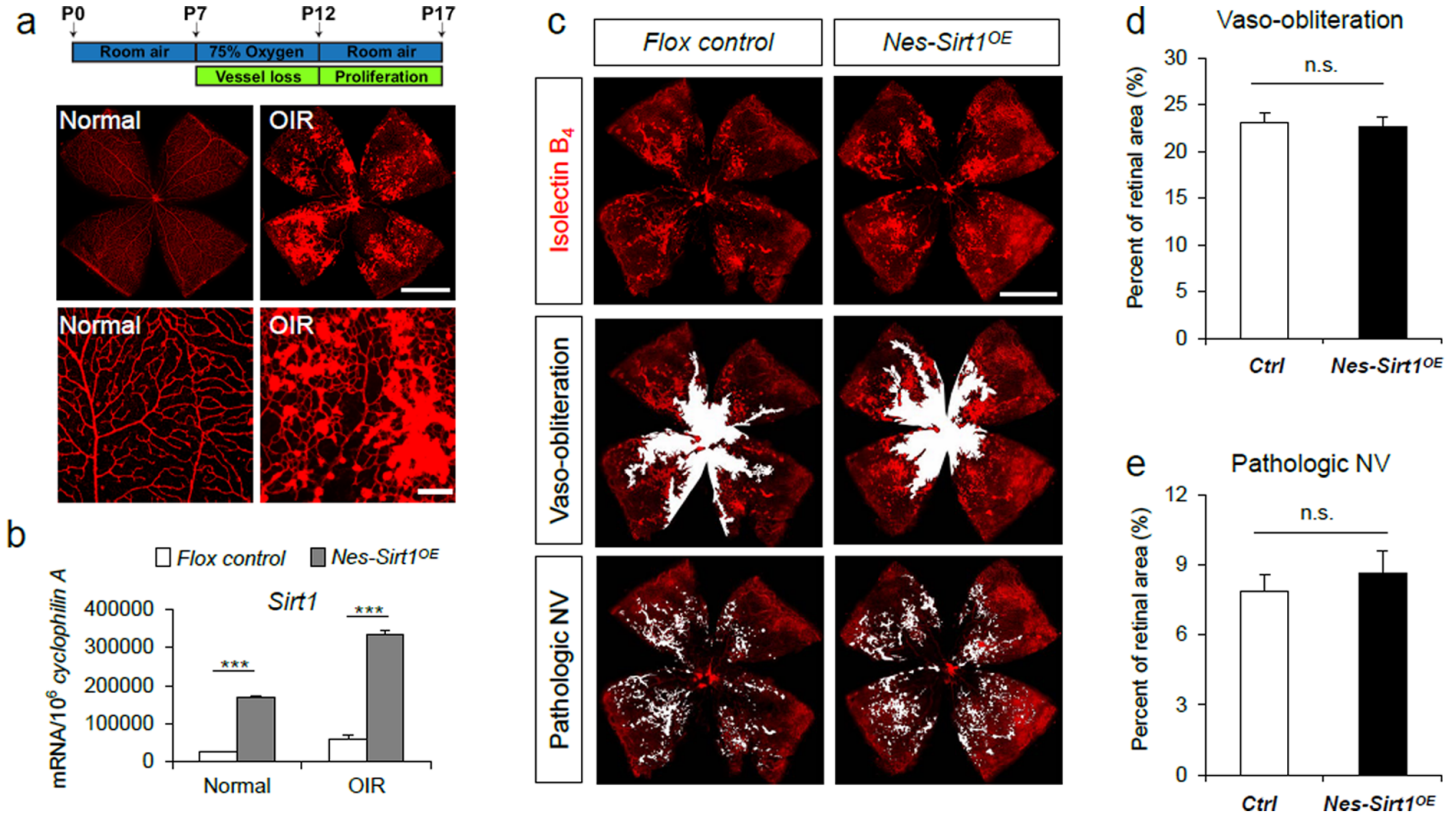
Sirt1 over-expression in retinal neurons does not protect against vascular degeneration in oxygen-induced retinopathy (OIR). a) Schematic illustration of OIR and representative images of retinal flat-mount with OIR and normoxic control retina at P17. To induce retinopathy, mice were exposed to 75% oxygen from P7 to P12. Retinas were dissected at P17 and stained with Isolectin B_4_ (red) to visualize vessels. Lower panels show enlarged areas of normal vessels in control retina and abnormal pathologic neovascularization in OIR retina. Scale bars: top panels: 1000 µm. Bottom panels: 150 µm. b) Increased Sirt1 levels in *Nes-Sirt1^OE^* retinas compared to control retinas, both in normal room air and with induced OIR. c) Representative images of retinal vasculature from *Nes-Sirt1^OE^* and littermate controls with induced OIR at P17. Areas of vaso-obliteration(VO) or pathologic neovascularization (NV) were highlighted in white. d) Quantification of retinal vaso-obliteration in OIR as percent of total retinal areas in *Nes-Sirt1^OE^* retinas and littermate controls. e) Quantification of pathologic neovascularization in OIR as percent of total retinal areas in *Nes-Sirt1^OE^* retinas and littermate controls. n = 15–20/group. ***p<0.001; n.s.: not significant.

Having confirmed Sirt1 over-expression in *Nes-Sirt1^OE^* retinas, we then evaluated whether over-expression of Sirt1 in retinal neurons influences vascular degeneration in a mouse model of oxygen-induced retinopathy (**Fig. 2a**). Mouse pups were exposed to 75% oxygen from postnatal day 7 to 12 to induce retinopathy, with maximal neovascular response detected at P17 (**Fig. 2a**)[13], [28], [29]. Previously, we found that Sirt1 is significantly upregulated in oxygen-induced retinopathy and conditional depletion of Sirt1 in retinal neurons significantly dampens vascular regrowth and precipitates pathologic neovascularization in retinopathy[12], suggesting that endogenous Sirt1 induction is important for protecting against retinopathy development. In this study we asked whether over-expression of Sirt1 may be protective in retinopathy. We first confirmed that in OIR, Sirt1 expression in flox control is increased compared to normoxic controls, and *Nes-Sirt1^OE^* retinas in OIR showed additional significantly upregulated levels of Sirt1 compared to littermate flox control retinas in OIR (**Fig. 2b**). Neuronal Sirt1 over-expression, however, does not significantly influence the levels of vaso-obliteration in OIR compared to littermate flox controls (control: 23.02±1.05% vs. *Nes-Sirt1^OE^*: 22.60±1.09%, n = 15–20/group, p = 0.79, **Fig. 2c,d**). There is also no significant difference in pathologic neovascularization (control: 7.89±0.70% vs. *Nes-Sirt1^OE^*: 8.62±0.48%, n = 15–20/group, p = 0.38, **Fig. 2c,e**), suggesting that increasing Sirt1 levels in retinal neurons is not advantageous in protecting against oxygen-induced retinopathy.

### Neuronal over-expression of Sirt1 does not impact retinal neuron degeneration in retinopathy

**Figure 3:**
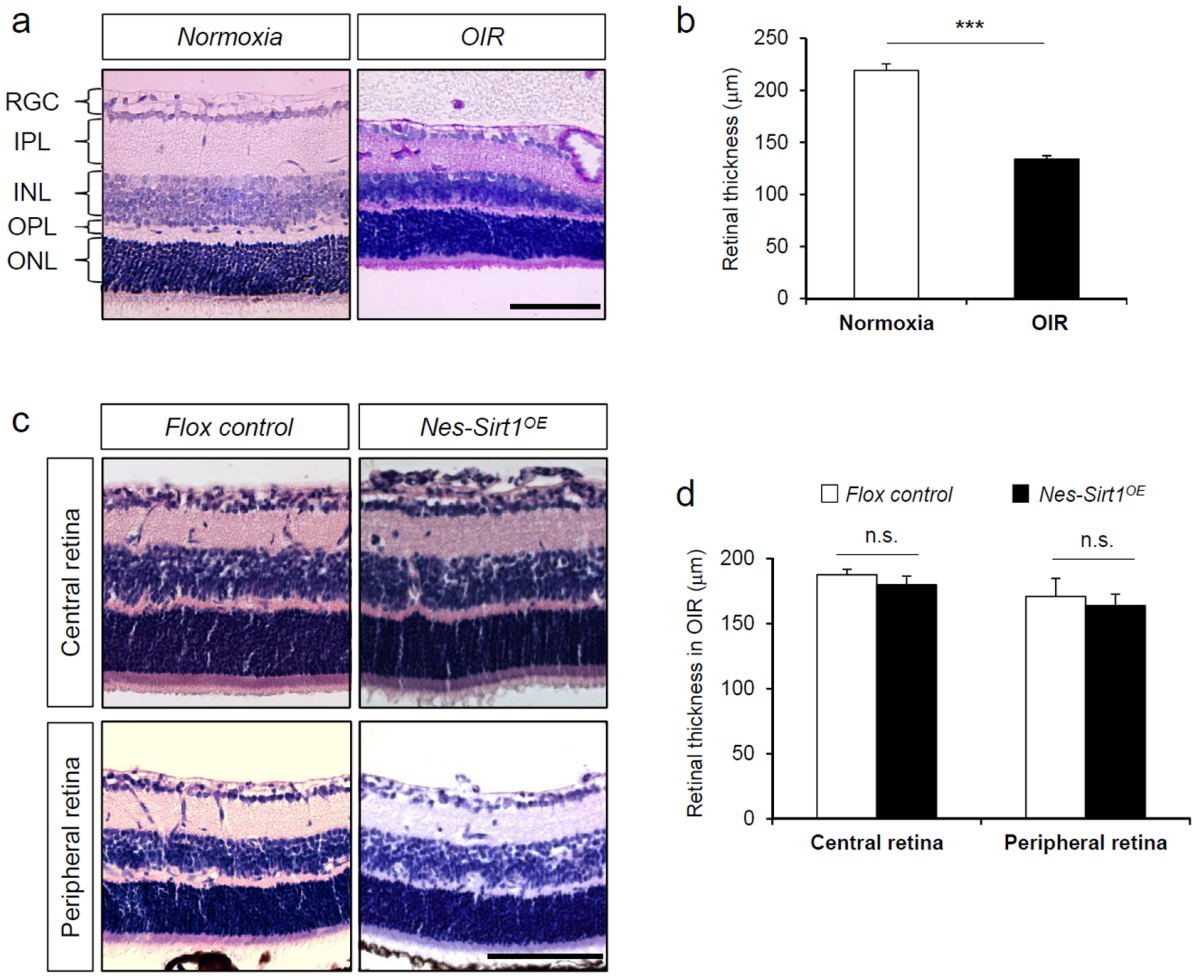
Neuronal overexpression of Sirt1 does not protect against neuronal degeneration in OIR. a) Representative images of retinal cross sections from OIR retinas and age-matched normoxic control retinas at P17. Paraffin embedded retinal cross sections were stained with H&E to visualize cellular structures. b) Total retinal thickness was quantified from OIR retinas and compared to normoxic control retinas. n = 6/group. c) Representative images of retina cross sections from *Nes-Sirt1^OE^* mouse and littermate flox control mice at P17 after OIR. *Nes-Sirt1^OE^* and littermate control mice were exposed to 75% oxygen from P7 to P12 to induce retinopathy. Retinas were dissected at P17 and paraffin embedded sections were stained with H&E to visualize cellular structure. d) Quantification of total retinal thickness from OIR exposed *Nes-Sirt1^OE^* mice and littermate flox control mice. n = 6/group. ***p<0.001; n.s.: not significant. Scale bars: 100 µm.

In addition to the vascular analysis, we assessed the impact of neuronal Sirt1 over-expression on retinal neuronal degeneration in OIR by measuring retinal thickness in paraffin embedded retinal cross sections. Normally there is significant retinal neuronal degeneration in OIR retinas after oxygen exposure, compared to age matched control mice raised in room air. At P17, total retinal thickness in OIR retinas is reduced approximately 30% compared to normoxia controls (normoxia: 219.15±6.34 µm vs. OIR: 133.81±3.46 µm, n = 6/group, p<0.0001, **Fig. 3a, b**). Most retinal thinning in OIR retinas occurs at the inner nuclear layer (INL), inner plexiform layer (IPL), and to a lesser extent in photoreceptor layer (**Fig. 3a, b**). On the other hand, *Nes-Sirt1^OE^* retinas in OIR do not show a significant difference in total retinal thickness at P17 compared to littermate flox controls in OIR in both central and peripheral areas of retina (**Fig. 3c, d**). These data indicate that Sirt1 over-expression in retinal neurons does not show detectable protection against neuronal degeneration in retinopathy as assessed by morphology.

### Conditional over-expression of Sirt1 in vascular endothelial cells does not protect against oxygen-induced retinopathy

**Figure 4:**
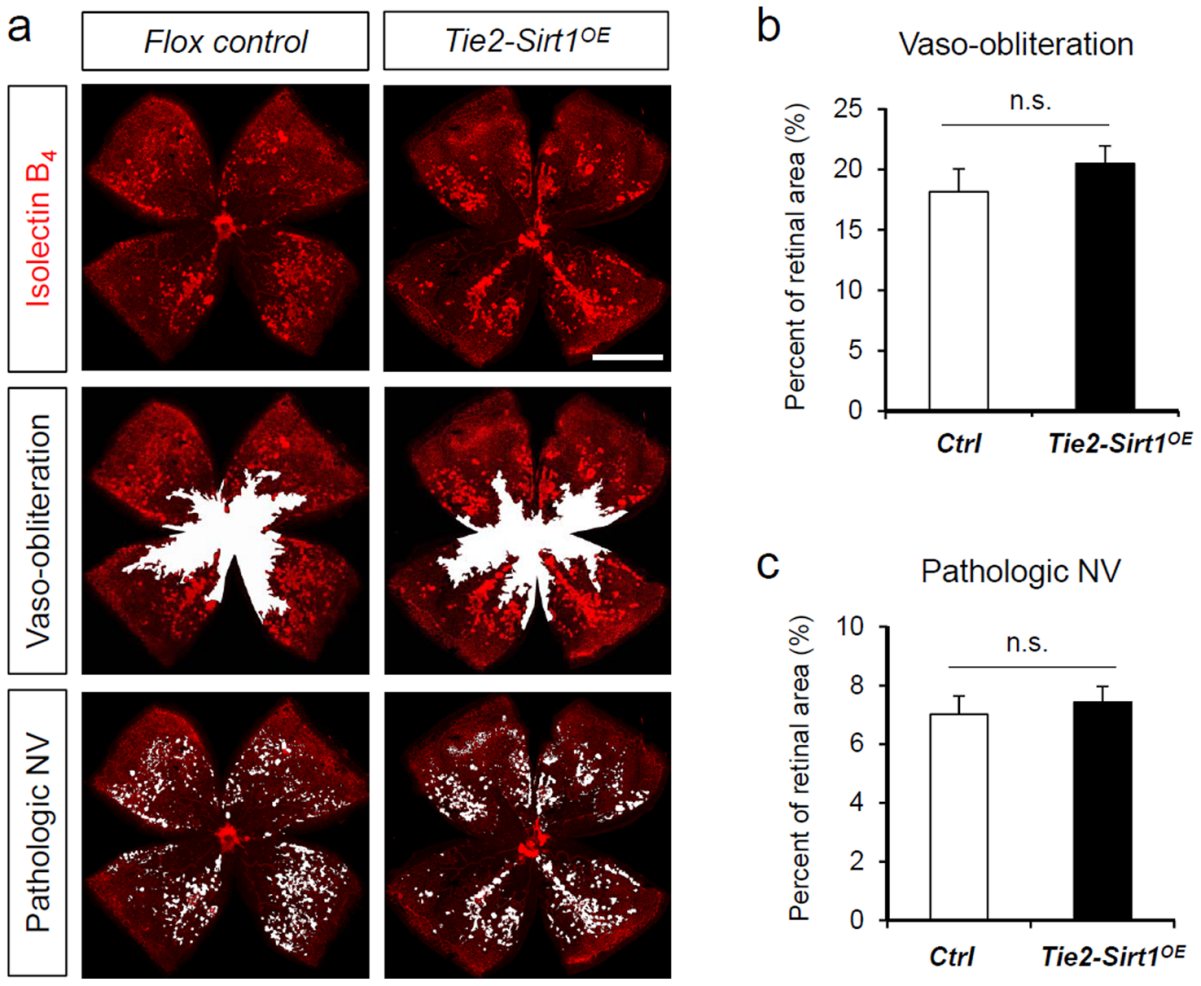
Sirt1 over-expression in retinal vascular endothelial cells does not protect against vascular degeneration in OIR. *Tie2-Sirt1^OE^* and littermate control mice were exposed to 75% oxygen from P7 to P12 to induce retinopathy. Retinas were dissected at P17 and stained with Isolectin B_4_ to visualize vessels (red). a) Representative images of retina flat-mounts from *Tie2-Sirt1^OE^* mice and littermate controls at P17 after OIR. Areas of vaso-obliteration(VO) or pathologic neovascularization (NV) were highlighted in white. Scale bar: 1000 µm. b) Quantification of retinal vaso-obliteration in OIR as percent of total retinal areas in *Tie2-Sirt1^OE^* and littermate controls. c) Quantification of pathologic NV in OIR as percent of total retinal areas in *Tie2-Sirt1^OE^* and littermate controls. n = 8–20/group; n.s.: not significant.

Previous studies showed that Sirt1 is important for sprouting angiogenesis, and endothelial specific knockout of Sirt1 impairs developmental angiogenesis[24], [25]. In addition to generating Nestin-driven Sirt1 over-expressor mice, we also generated endothelial specific Sirt1 over-expression mice (*Tie2-Sirt1^OE^*) by crossing Sirt1 flox mice with Tie2-Cre mice. Tie2-driven protein expression in the retina was previously localized specifically in the vasculature using Tie2-Cre reporter mice[34]. However, with induced OIR, conditional over-expression of Sirt1 in *Tie2-Sirt1^OE^* mice does not impact levels of vaso-obliteration compared to littermate flox controls (control: 18.17±1.88% vs. *Tie2-Sirt1^OE^*: 20.49±1.46%, n = 8–20; p = 0.36; **Fig. 4a, b**). Pathologic neovascularization is also not affected in *Tie2-Sirt1^OE^* retinas in OIR (control: 7.02±0.62% vs. *Tie2-Sirt1^OE^*: 7.42±0.55%, n = 8–20; p = 0.67; **Fig. 4a, c**). Together these results indicate that over-expression of Sirt1 in Tie2-expression vascular endothelial cells does not significantly impact vascular loss and pathologic neovascularization in OIR.

### Resveratrol treatment does not protect against oxygen-induced retinopathy

**Figure 5:**
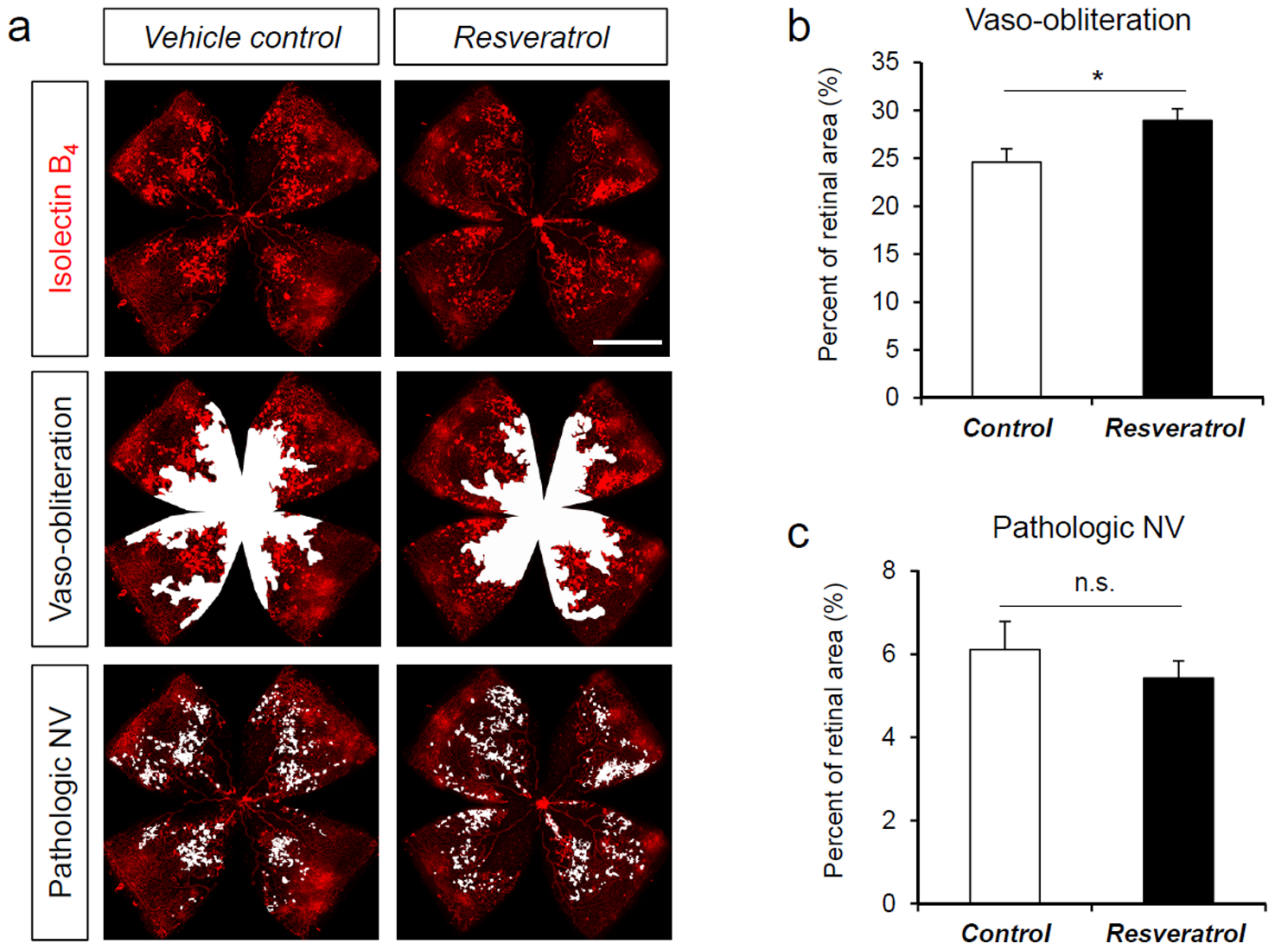
Resveratrol treatment does not suppress vascular pathologies in OIR. C57Bl/6 mouse pups were exposed to 75% oxygen from P7 to P12 to induce retinopathy. Littermate mouse pups were treated with resveratrol or vehicle control through daily oral gavage from P5 to P17. Retinas were dissected at P17 and stained with Isolectin B_4_ to visualize vessels. a) Representative images of retina flat-mounts from resveratrol treated mice and littermate vehicle controls at P17 after OIR. Areas of retinal vaso-obliteration(VO) in OIR and pathologic neovascularization(NV) were highlighted in white. Scale bar: 1000 µm. b) Quantification of vaso-obliteration as percent of total retinal areas in resveratrol treated mice compared to littermate controls. c) Quantification of pathologic NV in OIR as percent of total retinal areas in resveratrol treated mice compared to littermate controls. n = 15–18/group; *p<0.05; n.s.: not significant.

To complement the genetic approaches of over-expressing Sirt1 in transgenic mice, we assessed the effect of a Sirt1 activator, resveratrol, in OIR. Resveratrol is a natural phenol from plant extracts, and binds Sirt1 through allosteric binding[27]. Resveratrol was shown to improve the health and survival of high fat fed mice[35]. C57Bl/6 mice pups in OIR were treated with oral gavage of resveratrol (400 mg/kg body weight, daily) from P5 to P17. Compared to littermate mice fed with vehicle control, mice fed with resveratrol showed a modest yet significant increase of vaso-obliteration (control: 24.60±1.39% vs. resveratrol: 28.93±1.24%, n = 15–18/group, p = 0.015, **Fig. 5a,b**). The level of pathologic neovascularization is not significantly different, although there is a trend toward suppressing neovascularization with resveratrol (control: 6.10±0.68% vs. resveratrol: 5.43±0.41%, n = 15–18/group, p = 0.19, **Fig. 5a,c**). These results suggest that resveratrol does not protect against vascular pathologies in OIR at the administrated dose. The significantly increased VO may reflect a Sirt1 independent effect of resveratrol in directly suppressing vascular growth.

### Treatment with Sirt1 activator SRT1720 does not suppress pathologic neovascularization in oxygen-induced retinopathy

**Figure 6:**
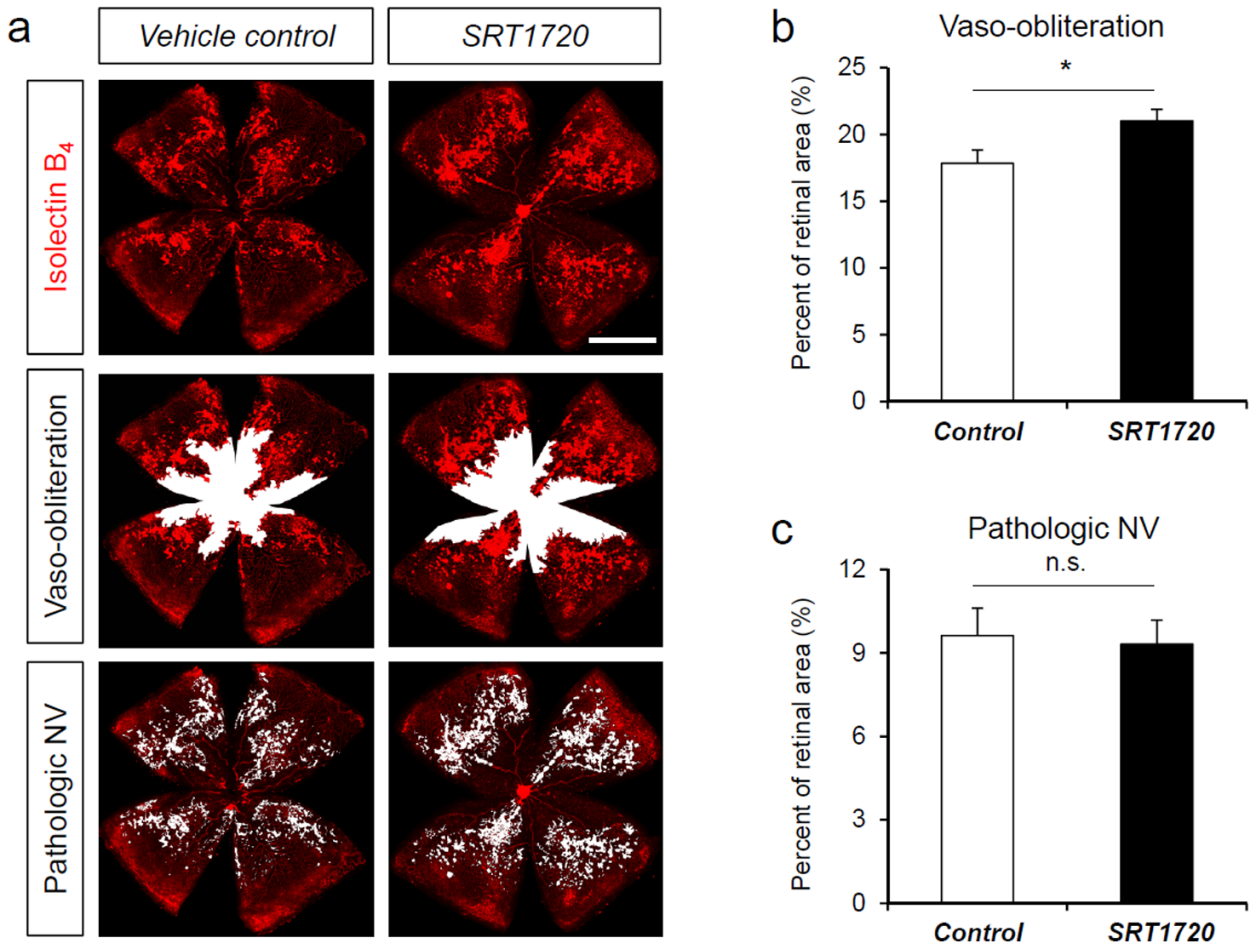
Sirt1 activator SRT1720 does not suppress vascular pathologies in OIR. C57Bl/6 mouse pups were exposed to 75% oxygen from P7 to P12 to induce retinopathy. Littermate mouse pups were treated with SRT1720 or vehicle controls through daily oral gavage from P5 to P17. Retinas were dissected at P17 and stained with Isolectin B_4_ to visualize vessels. a) Representative images of retina flat-mounts from SRT1720 treated mice and littermate vehicle controls at P17 after OIR. Areas of vaso-obliteration(VO) or pathologic neovascularization(NV) were highlighted in white. Scale bar: 1000 µm. b) Quantification of retinal vaso-obliteration in OIR as percent of total retinal areas in SRT1720 treated mice compared to littermate controls. c) Quantification of pathologic NV in OIR as percent of total retinal areas in SRT1720 treated mice compared to littermate controls. n = 18–20/group; *p<0.05; n.s.: not significant.

We next assessed the effect of SRT1720, a highly potent Sirt1 specific activator, in retinopathy. SRT1720 was shown previously to specifically activate Sirt1 to improve metabolic disorders in obese mice [26], [36]. In this study, C57Bl/6 mouse pups in OIR were treated with SRT1720 by oral gavage (100 mg/kg body weight, daily) from P5 to P17 (**Fig. 6a**). Compared to littermate mice fed with vehicle control, mice fed with SRT1720 showed a modest but significant increase of vaso-obliteration (control: 17.83±0.99% vs. Sirt1 OE: 21.01±0.86%, n = 19–20/group, p = 0.02, **Fig. 6a,b**), suggesting a detrimental effect of increased vessel loss. No significant difference in pathologic neovascularization was observed (control: 9.62±0.45% vs. SRT1720: 9.31±0.51%, n = 19–20/group, p = 0.46, **Fig. 6a,b**). These results suggest that Sirt1 activator SRT1720 at the administrated dose does not protect against vascular pathologies in OIR.

## Discussion

In contrast to our previously published data that show marked effects in retinopathy associated with the lack of Sirt1[12], our results here reveal that a genetically increased dosage of Sirt1 in either neurons or vessels within the mouse retina does not alter vaso-obliteration, pathologic neovascularization, or neuronal degeneration in oxygen-induced retinopathy. Furthermore, treatment with Sirt1 activators resveratrol or SRT1720, did not show protective effects against the development of retinopathy. Studies in different mouse models of human diseases have shown that while constitutive high levels of sirtuin overexpression (8 to 16 fold) may cause null or negative effects, inducing moderate amounts of sirtuins in tissues (lower than 7.5-fold) may confer protective effects. For instance, we have found previously that a ∼16-fold increase in Sirt1 in hippocampal mouse neurons does not have any effect in synaptic plasticity, learning or memory[20], whereas in the heart it was reported that an increase of Sirt1 above 8-fold may cause a negative effect in cardiac function[37]. In contrast, a moderate overexpression (∼4-fold) in cortical neurons confers a youthful gene transcription profile in old murine brains[38], and three to four-fold Sirt1 increase in the intestines of colon cancer prone mice[39], or in bone marrow lymphocyte progenitors of p53+/– lymphoma prone mice[38], protect both models from cancer. In addition, mice producing about twice as much brain Sirt1 protein as control mice show less anxiety and depression-like phenotypes[32], with decreased production of β-amyloid and plaques in a mouse model of Alzheimer's disease[21], and are protected against α-synuclein aggregation in a genetic mouse model of Parkinson's disease[23]. Although the overexpression system used herein allowed the transgenic Sirt1 mice to reach the desired moderate increase of Sirt1 (∼4-fold) in neuronal retina, a level similar to those that produced a protective effect in other mouse tissues, it was not optimal to alter the course of retinopathy. Strikingly, most of neuroprotective effects seen *in vivo* by Sirt1 overexpression have resulted from a mere 2-fold upregulation. Thus, potentially very low levels of NAD+ in the nervous system resulting from an overexpression of above 4-fold may offset Sirt1 effects in OIR. In addition, in OIR, the endogenous retinal Sirt1 level is highly upregulated[12], therefore increasing the protein level of Sirt1 with over-expression of ∼4-fold, may not result in additional gain in enzymatic catalytic activity of lysine deacetylaion to exert a protective effect. Moreover, endogenous upregulation of Sirt1was observed mostly in retinal ganglion cells[12], the neuronal cell type most closely associated with superficial layer of retinal vessels impacted in retinopathy, while as over-expression of Nestin-driven Sirt1was found in pan-retinal neurons, most significantly observed in inner nuclear layer composed mainly of bipolar cells. This difference of expression in cell specificity may also account for the lack of protective effects in Nes-Sirt1 mice, and suggest that cell specific expression of Sirt1 may play a role in determining directional regulation of blood vessel growth in retinopathy.

It is noteworthy that lysine acetylation of proteins is a transiently regulated mechanism in response to rapid and recurrent changes in tissue metabolic conditions. We have formerly reported that Sirt1 is highly induced in OIR, and proposed that Sirt1 promoted vascular regrowth in part by controlling the expression of angiogenic factors through mediating deacetylation and stability of hypoxia-induced factor (HIF) 1α and 2α[12]. Thus, a constitutively high level of Sirt1, as opposed to a metabolically regulated transient induction in response to environmental need, might alter acetylation levels of key factors involved in angiogenesis and other cellular processes, thereby hampering any Sirt1-dependent acute transient regulation in OIR. At present, thousands of proteins have been identified to be regulated by acetylation and among those hundreds are relevant for normal physiology, aging and pathology of the brain, and are targets of Sirt1[19]. However, limited information is known about protein acetylation in retinopathy and eye diseases. Recently, acetylation of retinal histones was found to increase inflammation in diabetic retinopathy[40], and overexpression of histone deacetylase 4 (HDAC4) was found to promote neuronal survival and protect against retinal degeneration[41]. Further studies will allow us to gain deeper insights in the role of protein acetylation and its regulation by sirtuins and other deacetylases in retinal diseases.

In contrast to limited *in vivo* studies of Sirt1 mutant mice in retinal diseases, small molecule Sirt1 activators have been evaluated in the eye in several studies. Since their discovery, Sirt1 activators have been investigated in various animal models and diseases including cancer, cardiovascular diseases and neurodegeneration, involving various drug forms and dosages[42]. While many have shown significant effects, others have none. One of the most challenging aspects of evaluating resveratrol's efficacy is the wide variety of available formulations, dosages, as well as routes and times of administration, all of which greatly influence the experimental results[43]. Among the few studies performed in the retina, it was found that resveratrol suppressed light-induced retinal degeneration by decreasing outer nuclear layer apoptosis and thinning, thereby preserving visual function as detected by electroretinography[44]. In human retinal pigment epithelium, resveratrol reduces oxidative stress and hyperproliferation[45]. In addition, a study in a rat model of oxygen-induced retinopathy showed that resveratrol modulates nitric oxide synthase, however the phenotypic effect on *in vivo* retinopathy was not thoroughly characterized[46]. In this previous study, 30 mg/kg resveratrol, normally administered orally, was injected intravitreally. The results differed compared to our experiments, where mice treated with oral gavage of 400 mg/kg resveratrol did not show significant protection in OIR. Interestingly, our study of resveratrol treatment through oral gavage showed a modest detrimental effect of increased vaso-obliteration, which may reflect a direct anti-angiogenic effect of resveratrol. This was also shown in our previous study in which oral resveratrol treatment in *Vldlr^−/−^* mice suppressed pathologic subretinal neovascularization[47], as well as in a separate study where resveratrol suppressed laser-induced choroidal neovascularization in a Sirt1 independent manner[48], suggesting that the specific role of resveratrol is likely disease and pathology dependent. In addition, a recent chemical screening study indicates that resveratrol is an inhibitor of SOCS3(suppressor of cytokine signalling 3) expression[49], depletion of which in vascular endothelial cells negatively impacts retinopathy[34]. This negative regulation of resveratrol on SOCS3 may also contribute in part to the lack of its protective effect in OIR.

Compared to resveratrol, SRT1720, a potent Sirt1 activator, has not been as thoroughly tested. Nonetheless, existing data on SRT1720 show that it produces diverse effects in multiple diseases, such as improving health and survival in obese mice and thereby implicated as a potential therapeutic for type 2 diabetes[26], [36]. SRT1720 has also been shown to suppress inflammation in a mouse model of asthma[50], and promote tumor cell migration and metastasis of breast cancer in mice[51]. However, the effects of SRT1720 in the nervous system and in the retina, have been scarcely addressed. Although our data did not show SRT1720 or resveratrol providing protection in retinopathy, a study in a mouse model of multiple sclerosis revealed that oral administration of this drug prevents axonal loss of retinal ganglion cells and optic nerve in optic neuritis, as did resveratrol[52].

While our data demonstrate that Sirt1 over-expression in the retinas increase the expression of Sirt1 target genes, whether induction of Sirt1 with a genetic approach or pharmacologic treatment indeed leads to increased Sirt1 enzymatic activity and hence physiologic function, remains undetermined due to technical challenges. Measuring Sirt1 enzymatic activity in tissue extraction, particularly in small samples such as the retina, is difficult. Fluorescent Sirt1 enzymatic assay has potential artifacts and thereby considered not reliable by most researchers[53]. Measuring Sirt1 reaction products O-acetyl adenosine diphosphate ribose by mass spectrometry, or nicotinamide with nicotin- amidase PNC1 and ortho-phthalaldehyde (OPT)[27] are not only technically difficult, but so far both assays have only been carried out with immunopurified protein mixed with externally provided substrates. Yet, in living tissue, the physiological regulation of Sirt1 relies on allosteric regulation of Sirt1, NAD^+^ bioavailability, and/or nicotinamide levels in specific cellular compartments, all of which are lost in homogenized cellular extraction. In addition, whole tissues like retinas, potentially express all seven isoforms of Sirtuins, which may potentially interfere with the Sirt1 enzymatic assay. Future development of more reliable, accurate Sirt1 activity assay for tissue extracts will help overcome these technical limitations and greatly benefit research in Sirt1's biologic activity.

In conclusion, while lack of Sirt1 promotes retinopathy in mouse OIR as we previously reported[12], the current study with Sirt1 genetic overexpression or oral treatment with gavaged Sirt1 activating compounds such as resveratrol or SRT1720 does not support their additive protective effect in retinopathy. Further studies evaluating different levels of Sirt1 overexpression, induction of this sirtuin in specific cell types, diverse activators' concentrations and routes of administration, as well as assessing the resultant Sirt1 enzymatic activity may allow us to examine more in depth whether increasing levels of Sirt1 may serve as a potential therapeutic approach to treat or prevent retinopathy.
